# Performance and Health Decrements Associated With Relative Energy Deficiency in Sport for Division I Women Athletes During a Collegiate Cross-Country Season: A Case Series

**DOI:** 10.3389/fendo.2021.524762

**Published:** 2021-05-12

**Authors:** David R. Hooper, Jared Mallard, Jeff T. Wight, Kara L. Conway, George G.A. Pujalte, Kelsey M. Pontius, Catherine Saenz, Anthony C. Hackney, Adam S. Tenforde, Kathryn E. Ackerman

**Affiliations:** ^1^ Department of Kinesiology, Jacksonville University, Jacksonville, FL, United States; ^2^ Department of Biology, Jacksonville University, Jacksonville, FL, United States; ^3^ Departments of Family Medicine, and Orthopedics and Sports Medicine, Mayo Clinic Florida, Jacksonville, FL, United States; ^4^ Meteor Nutrition, Jacksonville, FL, United States; ^5^ Department of Exercise and Sport Science, University of North Carolina at Chapel Hill, Chapel Hill, NC, United States; ^6^ Spaulding National Running Center, Department of Physical Medicine and Rehabilitation, Spaulding Rehabilitation Hospital, Harvard Medical School, Cambridge, MA, United States; ^7^ Neuroendocrine Unit, Massachusetts General Hospital and Harvard Medical School, Boston, MA, United States; ^8^ Divisions of Sports Medicine and Endocrinology, Boston Children’s Hospital, Boston, MA, United States

**Keywords:** relative energy deficiency in sport, female athlete triad, body composition, endurance athletes, resting metabolic rate, iron, vitamin D

## Abstract

The purpose of this case series was to evaluate the presence of low Energy Availability (EA) and its impact on components of Relative Energy Deficiency in Sport (RED-S) in a population of female collegiate runners. Seven female NCAA Division I athletes (age: 22.3 ± 1.5 yrs; height: 169.7 ± 5.7 cm; weight: 58.3 ± 4.1 kg) were tracked from August until February, covering the beginning (Pre XC), end (Post XC) of their competitive cross country season, and beginning of the following track season (Pre Track). The athletes were assessed for female athlete triad (Triad) risk, energy availability, body composition, resting metabolic rate (RMR), nutritional intake, and blood markers (including vitamin D, ferritin, and triiodothyronine (T3)). From Pre XC to Post XC there were no significant differences in body mass, fat free mass or body fat percentage. At Pre XC, mean EA was 31.6 ± 13.3 kcal/kg FFM∙d^-1^. From Post XC to Pre Track, there was a significant increase in body mass (59.1 ± 5.1 to 60.6 ± 5.7 kg, p<0.001,d=0.27). From Post XC to Pre Track, there was a significant increase in RMR (1466 ± 123.6 to 1614.6 ± 89.1 kcal·d^-1^, p<0.001,d=2.6). For 25(OH) vitamin D, there was a significant reduction from Pre XC to Post XC (44.1 ± 10.6 *vs* 39.5 ± 12.2 ng·mL^-1^, p=0.047,d=-0.4), and a significant increase from Post XC to Pre Track (39.5 ± 12.2 *vs.* 48.1 ± 10.4 ng·mL^-1^, p=0.014,d=0.75). For ferritin, there was a trend towards a decrease from Pre XC to Post XC (24.2 ± 13.2 *vs.* 15.7 ± 8.8 ng·mL^-1^, p=0.07, d=-0.75), as well as a trend toward an increase from Post XC to Pre Track (15.7 ± 8.8 *vs.* 34.1 ± 18.0 ng·mL^-1^, p=0.08, d=1.3). No differences in T3 were observed across time points. Average Triad risk score was 2.3 ± 1.4. Notably, 5 of 7 athletes met criteria for moderate risk. Despite many athletes meeting criteria for low EA and having elevated Triad risk assessment scores, most were able to maintain body mass and RMR. One athlete suffered severe performance decline and a reduced RMR. Surprisingly, she was the only athlete above the recommended value for ferritin. Following increased nutritional intake and reduced training volume, her performance and RMR recovered. Changes in body mass and body composition were not indicative of the presence of other concerns associated with RED-S. This exploratory work serves as a guide for future, larger studies for tracking athletes, using RMR and nutritional biomarkers to assess RED-S.

## Introduction

The consequences of low energy availability (EA) in female endurance athletes have now been known for decades, which initially included menstrual dysfunction and a reduction in bone mineral density (BMD). The interrelationship of these factors became known as the Female Athlete Triad (Triad) with the American College of Sports Medicine (ACSM) publishing position stands in 1997 (1) and 2007 (2) to help clinicians recognize, treat, and prevent these clinical conditions. More recently, a new term, Relative Energy Deficiency in Sport (RED-S) was proposed (3, 4) as an extension of Triad, with the intent to additionally include male athletes, as well as many other aspects of impaired physiological function, including metabolic rate, immunity, protein synthesis, and cardiovascular health (4). Further, it was specifically stated that this clinical phenomenon of low EA is not only a ‘triad’ of three entities but a syndrome that may influence multiple aspects of health and performance (4).

EA is defined as energy intake (kcal/day) minus exercise energy expenditure (kcal/day), normalized to fat free mass (kg), thereby representing the energy available to support basic physiologic function outside exercise. Low EA has been defined as below 30 kcal·kg^-1^ of fat free mass per day after it was demonstrated that under this threshold, the negative health outcomes began to emerge (5) and is more common in athletes participating in endurance sports, such as running (6). Some suggest that 45 kcal·kg^-1^ of fat free mass per day may be optimal as energy intake for the expenditure expected in athletes (2, 7).

While less well studied, low EA may result in reductions of resting metabolic rate (RMR), and has been noted in both the RED-S (4) and Triad literature (7). In order to assess whether RMR has been reduced in anorexic women, studies have compared measured RMR to predicted RMR (RMRratio) utilizing established prediction equations, and have demonstrated ratios as low as 0.60-0.84 reported (8, 9). This practice was extended to exercising women, and those with a high drive for thinness demonstrated a RMRratio of 0.85, significantly lower than that of exercising women with a normal drive for thinness of 0.9 (10). Thus, it has now been suggested that a ratio less than 0.9 be used as a marker for low EA (7). This reduction in RMR is thought to be a reflection of adaptations that act as an energy-conserving mechanism, where the effects are translated to changes in metabolic rate (10).

One factor tied to metabolic rate in anorexic populations is triiodothyronine (T3), with changes in T3 showing associations with changes in RMR. This was demonstrated both by significantly lower T3 and resting energy expenditure (REE) when comparing underweight individuals with anorexia to normal weight women, as well as concomitant increases in T3 and REE as women with anorexia were treated and gained body mass (11). In exercising women with a high drive for thinness, significantly lower RMRratios were seen in conjunction with a reduced T3 when compared to exercising women without a high drive for thinness, as determined by the Eating Disorder Inventory (12). Thus, T3, in addition to RMR and RMRratio should be explored as a means of assessing adaptation to chronic energy deficiency (7). Collectively, these markers can be used to screen athletes to identify those who may be at risk for low EA or RED-S.

If low EA is occurring as a result of low total energy intake, naturally there may be concerns about meeting macronutrient and micronutrient recommendations. To provide adequate fuel for intense aerobic exercise, high quantities of carbohydrate intake are typically encouraged, with the International Olympic Committee recommending 6-10 g·kg^-1^·d^-1^ for moderate to high intensity exercise of 1-3 h·d^-1^ (13). In addition, to support recovery from exercise, adequate protein intake is also advised, with recommendations from ACSM ranging from 1.2-2.0 g·kg^-1^·d^-1^ (14). ACSM also advocates maintaining an intake of fat of at least 20 percent of overall intake, to meet the lower end of each macronutrient recommendation. As such, a 60-kg athlete would need to consume a minimum of 2,100 kcal·d^-1^. Additionally, in terms of micronutrients, impairment in iron status can reduce performance at serum ferritin concentrations of less than 25 ug·L^-1^ (15), which is frequent in endurance athletes (16). Low vitamin D is also a common concern in athletic populations, and is of particular importance in athletes with potential bone health issues due to its role in calcium regulation (17). In addition to bone, previous reports have shown detrimental effects on muscle function with 25(OH) vitamin D levels of less than 30 nmol·L^-1^ (12 ng·mL^-1^) (18). Ultimately, if overall energy intake is low, the consequences of low total calories, essential nutrients, and supporting macronutrient and micronutrient intake may lead to compromised fuel availability, recovery, oxygen transport and bone health, in addition to other concerns associated with low EA.

To date, limited prospective studies have been conducted in exercising women to define characteristics of low EA and changes to markers of metabolism and nutrition. Studies of performance in the setting of low-EA are also limited. Therefore, the purpose of this study was to evaluate the presence of low EA in a population of female collegiate runners with the consideration of each individual athlete in a case series approach. We hypothesized that components of RED-S would be observed in athletes with low EA, including markers of malnutrition (reduced ferritin, vitamin D status), suppressed RMR and observed reduced performance.

## Materials and Methods

**Figure 1:**
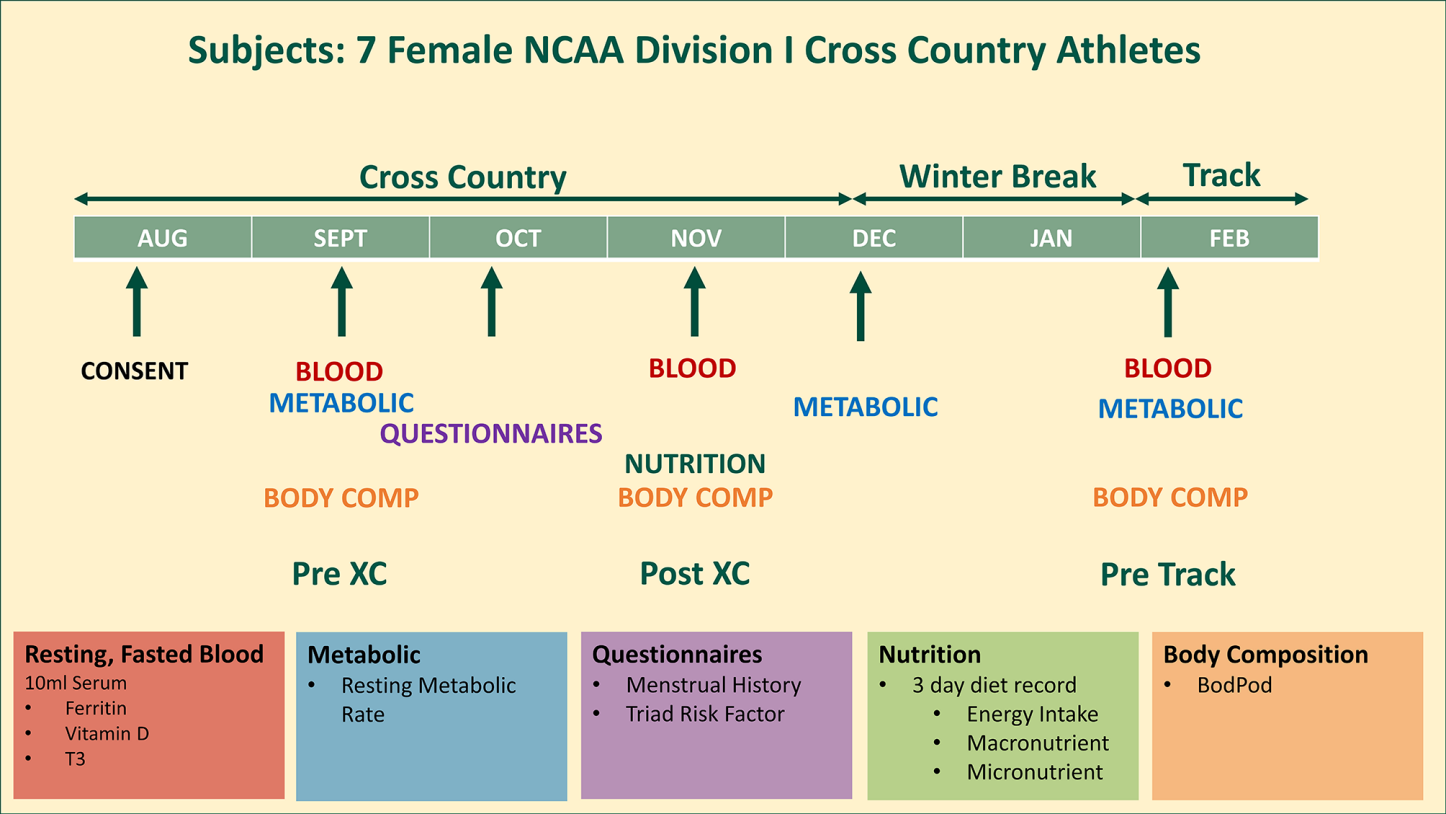
Study design. 7 female NCAA Division 1 collegiate cross country and track and field athletes complete testing before (Pre XC) and after (Post XC) cross country season, and before track (Pre Track) season.

An entire cross country team of seven female NCAA Division I distance runners (age: 22.3 ± 1.5 years; height: 169.7 ± 5.7 cm; weight: 58.3 ± 4.1 kg) were studied from August until February, covering the beginning (Pre XC) and end (Post XC) of their competitive cross country season, as well as the beginning of the subsequent track season (Pre Track). The athletes were assessed for a variety of factors that pertain to RED-S, including their Triad risk, metabolic rate, body composition, nutritional intake and blood markers (**Figure 1**). All athletes were fully informed of the protocol design and associated risks of this investigation before signing an informed consent document approved by Jacksonville University Institutional Review Board for use of human subjects.

### RED-S CAT

Athletes were scored according to the RED-S Clinical Assessment tool (19). Briefly, ‘red’ corresponds to an athlete who is high risk due to the presence of an eating disorder or other serious medical condition related to low EA. ‘Green’ is low risk, with appropriate physique and healthy eating habits. ‘Yellow’ is between the other two levels, characterized by factors such as abnormally low body fat, prolonged low EA, amenorrhea, menarche after age 15 years, low bone mineral density as well as others. These are athletes who may participate in exercise, but would benefit from monitoring.

### Triad Risk

A menstrual history questionnaire was used to formulate each individual athlete’s Triad Cumulative Risk Assessment score, which was interpreted as follows: 0-1 points = low risk; 2-5 points = moderate risk; ≥6 points = high risk (7). This assessment took place at the Pre XC time point.

### Resting Metabolic Rate

Athletes arrived at the Exercise Physiology Laboratory following an overnight fast and free from any strenuous physical activity in the last 24 hours. RMR was measured *via* indirect calorimetry using a Parvo TrueOne 2400 (Parvo Medics, Sandy, UT) metabolic cart. Athletes were supine with their heads covered fully with a canopy. Each testing session lasted 30 minutes, with the first 10 minutes of data removed from analyses and an average of the final 20 minutes of data collection used to produce a RMR. Further, any single minute average where the fraction of end tidal carbon dioxide (FECO_2_) concentration was not between 0.8 and 1.2 was removed from analyses. This measured RMR was compared to the predicted RMR as a means of assessing whether RMR was within the expected range (RMRratio) using the Harris-Benedict equation (20). Relative RMR, expressed as RMR per total body mass, was also calculated.

### Body Composition

Body composition, including fat mass (FM) and fat-free mass (FFM) was assessed by whole body densitometry using air displacement *via* the Bod Pod^®^ (Life Measurements, Concord, CA) in accordance with the manufacturer’s instructions. During testing, athletes wore only tight fitting clothing (e.g., swimsuit, single-layer compression shorts, or undergarments) and an acrylic swim cap. Body composition assessments took place at Pre XC, Post XC and Pre Track.

### Nutritional Intake

Nutrition intake was assessed by a 3-day diet record, with the athletes logging all food, drink, and supplement intake over the course of 2 weekdays and 1 weekend day at Pre XC. Subjects were encouraged to complete the logs during the day after each intake. The team dietitian reviewed the 3-day diet recall and met with team members individually to discuss their current intake from food and beverages vs. estimated energy needs. To estimate energy requirements to establish appropriate energy availability, the dietitian used calculated RMR plus an activity factor (based on student athlete reported activity) plus estimation of energy expended for training. If energy availability was deemed suboptimal (less than 45 kcal·kg^-1^ of fat free mass per day), the dietitian made recommendations that would allow the student athlete to achieve adequate EA. The nutrition logs were analyzed and reviewed by a registered dietitian using Nutritionist Pro software (Axxya Systems, Redmond, WA).

### Blood Markers

Athletes arrived at the Exercise Physiology Laboratory following an overnight fast. Blood was drawn from an antecubital vein by a trained phlebotomist into a serum vacutainer (10mL). The serum was then separated by centrifuge at 1500×g for 15 min and subsequently stored at -80 °C until it was analyzed in batch. Samples were thawed once only and analyzed in duplicate by enzyme-linked immunoassay (ELISA) (CALBiotech, Spring Valley, CA) for ferritin, 25(OH) vitamin D and T3, with sensitivities of 2.5 ng·mL^-1^, 2.5 ng·mL^-1^ and 0.05 ng·mL^-1^, respectively. All inter-plate and intra-plate coefficients of variance were under 10%.

### Statistical Analyses

In this case series, individual athlete data point changes were assessed by visual inspection and in the case of blood variables, compared to known reference ranges. All group data were assessed for normal distribution utilizing the Shapiro-Wilk method and all data were revealed to be normally distributed. Therefore, for each dependent variable, a repeated measures ANOVA was performed to assess changes in each of the dependent variables across time points (Pre XC, Post XC, Pre Track). When a significant ANOVA was reported, post-hoc comparisons were made using dependent t-tests with a Bonferroni correction factor applied to multiple comparisons. Missing data points were replaced with the mean value at the corresponding time point. Less than 10% of the data was replaced. For blood, if the measured value was below the detectable range for that particular assay, the lowest value within the detectable range was used. Statistical significance in this investigation was set at p≤ 0.05. To determine the magnitude of change, a Cohen’s d effect size was performed. The criteria used to interpret the magnitude of the effect size were 0.2 small, 0.5 medium, and 0.8 large (21). All data were analyzed using Statistical Package for the Social Sciences (version 25.0, IBM, Chicago, IL).

## Results

### RED-S CAT

The RED-S CAT classification for each athlete is shown in **Table 1**. Three athletes were categorized as ‘Green’ and 4 athletes were categorized as ‘Yellow’.

**Table 1:** Individual athlete RED-S Clinical Assessment Tool risk, menstrual status, Triad risk, Triad risk classification and energy availability of 7 NCAA Division 1 cross country and track and field athletes assessed prior to cross country season.

Athlete	RED-S CAT	Menses (In Past 12 Months)	Triad Risk	Triad Risk Classification	Energy Availability (kcal/kg FFM·d^-1^)
1	Green	≥ 12	0	Low Risk	Unable to obtain
2	Yellow	≥ 12	2	Moderate Risk	24.4
3	Yellow	9-11	3	Moderate Risk	25.5
4	Green	≥ 12	1	Low Risk	55.9
5	Green	6-8	3	Moderate Risk	29.4
6	Yellow	6-8	4	Moderate Risk	18.2
7	Yellow	0-2	3	Moderate Risk	36.3

Risk scores and classifications based on prior research (7, 19).

### Triad Risk

The Triad risk score and classification for each individual athlete, along with her respective EA is shown in **Table 1**. Average Triad risk score was 2.3 ± 1.4. Notably, 5 of 7 athletes met criteria for moderate risk. We were unable to obtain EA for 1 athlete. Four of the 6 athletes with known EA were below 30 kcal/kg-FFM/day.

### Resting Metabolic Rate

**Figure 2:**
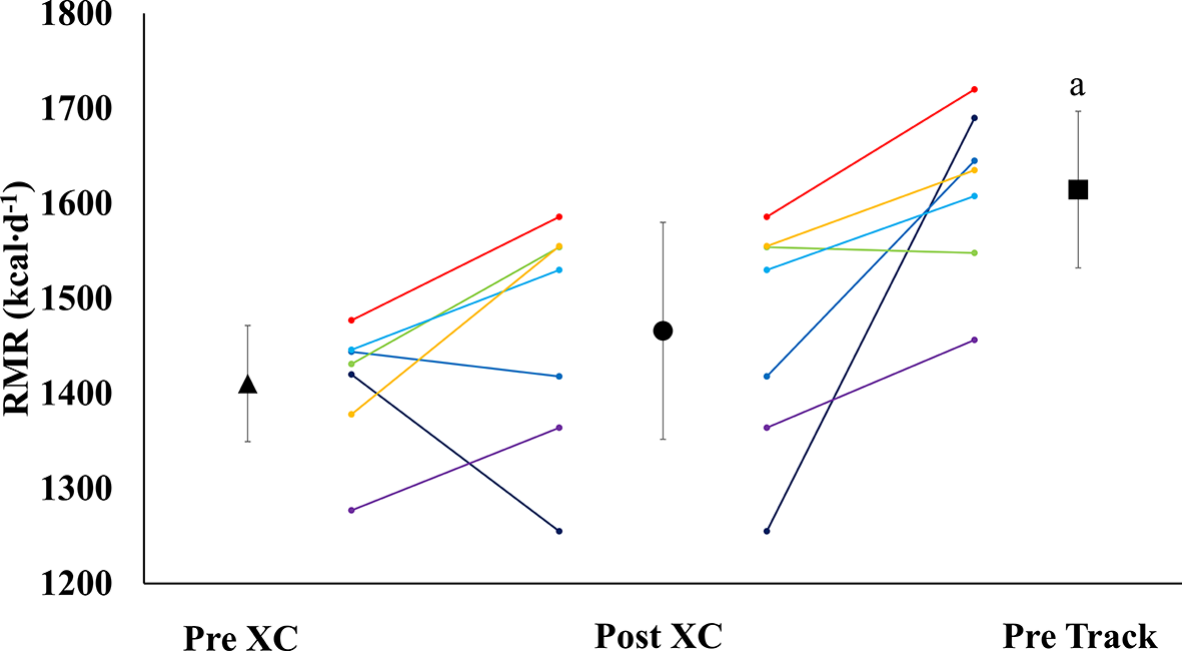
Resting Metabolic Rate (RMR) (mean ± 95% CI) of 7 female NCAA Division 1 collegiate cross country and track and field athletes before (Pre XC) and after (Post XC) cross country season, and before track (Pre Track) season. a= statistically significantly (p<0.05) different from Pre XC.

**Figure 3:**
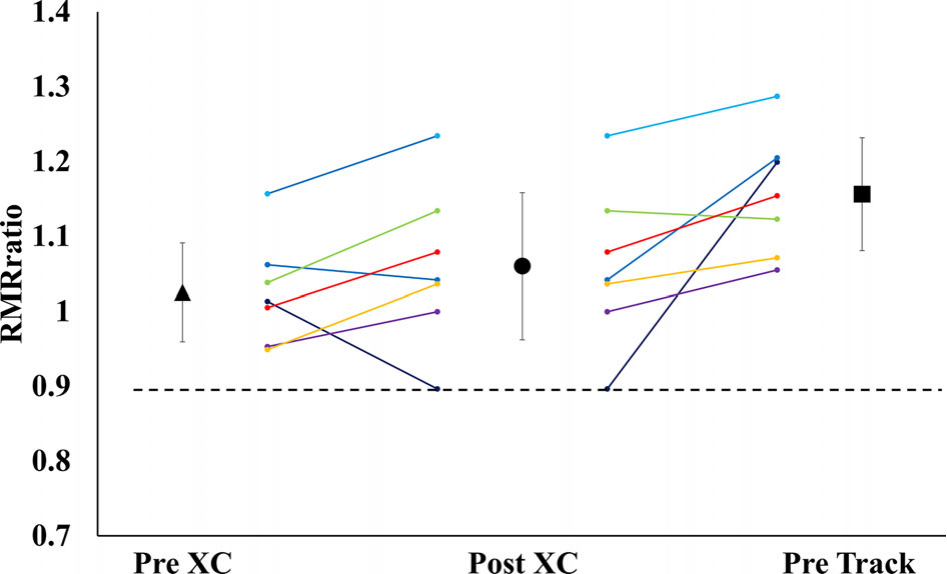
Measured Resting Metabolic Rate to Predicted Resting Metabolic Rate Ratio (RMRratio) (mean ± 95% CI) of 7 female NCAA Division 1 collegiate cross country and track and field athletes before (Pre XC) and after (Post XC) cross country season, and before track (Pre Track) season.

There were no significant differences in RMR from Pre XC to Post XC (Pre XC: 1410.4 ± 66.1 *vs.* Post XC: 1466.0 ± 123.6 kcal·d^-1^, p=0.248, d=0.56). There was a significant increase in RMR from Post XC to Pre Track (Post XC: 1466.0 ± 123.6 *vs.* Pre Track: 1614.6 ± 89.1 kcal·d^-1^, p<0.001, d=2.6) (**Figure 2**). There were no significant differences in RMRratio at any time point. Only one athlete was below the threshold of 0.9 at Post XC (**Figure 3**). There were no significant differences in relative RMR from Pre XC to Post XC (Pre XC: 24.3 ± 1.8 *vs.* Post XC: 24.9 ± 2.7 kcal·d^-1^·kg BM^-1^, p=0.39, d=0.27). There was a significant increase in relative RMR from Pre XC to Pre Track (Pre XC: 24.3 ± 1.8 *vs.* Pre Track: 26.8 ± 2.6 kcal·d^-1^-kg BM^-1^, p<0.001, d=1.13)

### Body Composition

**Figure 4:**
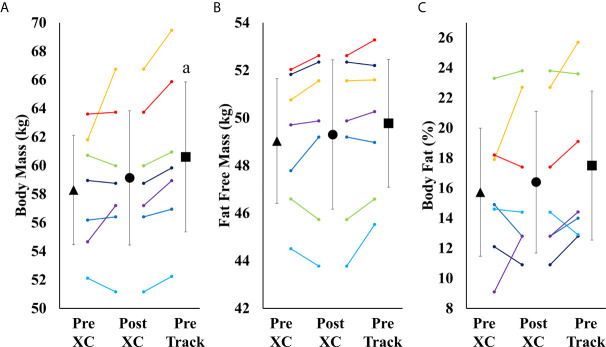
Body Mass **(A)**, Fat Free Mass **(B)** and Body Fat Percentage **(C)** (mean ± 95% CI) of 7 female NCAA Division 1 collegiate cross country and track and field athletes before (Pre XC) and after (Post XC) cross country season, and before track (Pre Track) season. a= statistically significantly (p<0.05) different from corresponding Pre XC.

There were no significant differences in body mass, fat free mass or body fat percentage from Pre XC to Post XC. There was a significant increase in body mass from Post XC to Pre Track (Post XC: 59.1 ± 5.1 *vs.* Pre Track 60.6 ± 5.7 kg, p<0.001, d=0.27) (**Figure 4**). There were no other significant differences in fat free mass or body fat percentage from Post XC to Pre Track.

### Nutritional Intake

Descriptive data pertaining to nutritional intake are displayed in **Table 2**. Athletes were below recommended carbohydrate intake, but were within protein recommendation guidelines.

**Table 2:** Nutritional intake of 7 NCAA Division 7 NCAA Division 1 cross country and track and field athletes assessed during competitive cross country season.

Variable	Mean (95% CI)	ACSM Recommendations
Kcal	2146 (1756–2535)	
EA (kcal-kg FFM-d^-1^)	32 (21–42)	>30 kcal/kg FFM/d Aim for 45 kcal/kg FFM/d
CHO (g)	202 (143–262)	
CHO (g·kg BM^-1^)	4.6 (3.5-5.6)	6-10 g/kg/d
Protein (g)	87 (74–100)	
Protein (g·kg BM^-1^)	1.5 (1.3-1.7)	1.2-2.0 g/kg/d
Fat (g)	50 (42–59)	
Fat (%)	13 (11–17)	20-35% total intake

Recommendations provided by American College of Sports Medicine (14).

### Blood

**Figure 5:**
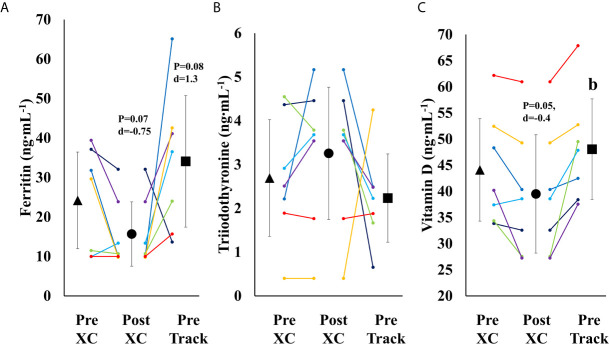
Serum Ferritin **(A)**, Triiodothyronine **(B)** and Vitamin D **(C)** (mean ± 95% CI) of 7 female NCAA Division 1 collegiate cross country and track and field athletes before (Pre XC) and after (Post XC) cross country season, and before track (Pre Track) season. b= statistically significantly (p<0.05) different from corresponding Post XC.

There was a trend towards a decrease in ferritin from Pre XC to Post XC (Pre XC: 24.2 ± 13.2 *vs.* Post XC: 15.7 ± 8.8 ng·mL^-1^, p=0.07, d=-0.75), as well as a trend towards increased ferritin from Post XC to Pre Track (Post XC: 15.7 ± 8.8 *vs.* Pre Track: 34.1 ± 18.0 ng·mL^-1^, p=0.08, d=1.3) (**Figure 5A**). There was a significant reduction in 25(OH) vitamin D from Pre XC to Post XC (Pre XC: 44.1 ± 10.6 *vs.* Post XC: 39.5 ± 12.2 ng·mL^-1^, p=0.047, d=-0.4). There was also a significant increase in 25(OH) vitamin D from Post XC to Pre Track (Post XC: 39.5 ± 12.2 *vs.* Pre Track 48.1 ± 10.4 ng·mL^-1^, p=0.014, d=0.75) (**Figure 5** **C**). There were no significant differences in T3 concentration at any time point.

### Performance

Performance level for each athlete is expressed as a percentage of her corresponding lifetime best performance in that particular event. These data are shown in **Table 3**.

**Table 3:** Performance level of each individual athlete during cross country and subsequent track seasons expressed as each athlete’s best performance of the corresponding season compared to their personal best time.

Athlete	XC Event	XC SR % of PR	Track Event	SR % of PR
1	5k	87.2	800	98.1
2	5k	105	800	100
3	5k	97.9	3000s	99.7
4	5k	96.6	1500	98.8
5	5k	N/A	N/A	N/A
6	5k	94.9	1500	N/A
7	5k	N/A	10k	N/A

XC, Cross Country; SR, Season Record; PR, Personal Record.

## Discussion

### Overview

The purpose of our investigation was to prospectively evaluate a team of seven collegiate cross-country runners, an endurance sport with elevated risk for low EA (22), and measure prospective changes in health and performance. Despite many athletes meeting criteria for low EA and having elevated Triad risk assessment scores, most were able to participate and maintain body mass and RMR throughout the season. Changes in body mass and body composition were not indicative of the presence of other concerns associated with RED-S, such as the observed reduced ferritin and 25(OH) vitamin D concentrations in-season. While both ferritin and 25(OH) vitamin D became reduced (trend) over the cross-country season, these values did appear to increase by the beginning of the following competitive season. These findings suggest that changes observed with low EA may be complex and not possible to evaluate with a single biomarker, and are likely highly individualistic.

### Case Observations

#### Performance Decline

The athlete with the worst in-season performance (Athlete 1, **Table 3**) showed severe reductions in performance during the cross country season, with her season record (SR) performance more than 2.5 minutes slower than her personal record in the 5km event, representing a 12% reduction in performance (**Table 3**). There was no obvious explanation for the notable decline in performance, however, the Head Coach decided to remove the athlete from cross country training and competition and she immediately began training for her track event, the 800m. This resulted in a substantial reduction in training volume. Following a meeting with the team dietitian, the athlete was also encouraged to increase caloric intake. All other athletes were within 5.1% of their respective personal records for the 5k event during the cross country season (**Table 3**).

#### Reduction in RMR

A proposed indicator of low EA is a reduction in RMR, which can be expressed as a ratio of measured RMR to predicted RMR (RMRratio); DeSouza et al. recommend a threshold of 0.9 as a marker for low EA (7). In the current study, the absolute RMR and RMRratio followed the same pattern as body mass from Pre XC to Post XC (i.e., no significant changes) for the group as a whole; although, Athlete 1 did demonstrate a RMRratio below 0.9 at Post XC (**Figure 3**). The removal of Athlete 1 from cross country training and competition, as previously mentioned, led to a drastic reduction in overall energy expenditure, combined with the recommendation from the team dietitian to increase energy intake, substantially increasing EA. Following the off-season, the athlete increased absolute RMR from 1255 to 1690 kcal·d^-1^ and increased RMRratio from 0.89 to 1.19. This athlete also had a much more successful track season compared to cross country, with a season-best within 3 seconds (2%) of her personal record (**Table 3**). While a case observation, these results show the potential ability of a reduced RMR to detect concerns related to RED-S, with a training and nutritional intervention affecting performance.

#### Blood Markers

Over the course of 9 weeks from Pre XC to Post XC, body mass and body composition were maintained (**Figure 4**), despite 4 of the 7 athletes at Pre XC demonstrating EA under the threshold of 30 kcal·kg^-1^ of fat free mass (**Table 1**) (2). Nonetheless, there were other concerns for these athletes related to their bone health and performance levels with respect to their blood markers. On average, there were significant reductions in 25(OH) vitamin D concentrations across time. This reduction from Pre XC to Post XC may have been a consequence of the subjects spending less time outdoors during the late fall into the winter months, although this would not explain why the vitamin D concentrations rebounded from Post XC to Pre Track, the time period corresponding to the coldest temperatures and least sun exposure in the region (December-January). A reduction in vitamin D is a significant concern, particularly for athletes susceptible to RED-S, as reduced bone mineral density is an established consequence of low EA (2) and vitamin D plays a critical role in bone health (17).

Ferritin levels were also below target for performance, as only one athlete maintained a ferritin concentration above the threshold of 25 ng·mL^-1^ (15) at Post XC. The group mean for ferritin was well below that threshold (15.7 ng·mL^-1^). A study of 165 female collegiate rowers found that athletes with a ferritin level above a threshold of 20 ng·mL^-1^ (n=44) had a statistically-significantly improved 2-km rowing time trial performance compared to those below the threshold (n=121) (15). Somewhat surprisingly, the one athlete in our study with a ferritin concentration above the recommended 25 ng·mL^-1^ was Athlete 1, who suffered the substantial performance decline. Ferritin concentration changes in this population were not indicative of changes in performance as has been previously shown, suggesting a complex interaction of multiple influences on performance.

### Group Observations

Following the cross country season, the athletes saw statistically significant increases in RMR (**Figure 2**), 25(OH) vitamin D (**Figure 5**), and a trend toward increases in ferritin (**Figure 5**) (p=0.08). Although efforts were made to reevaluate EA at this time, unfortunately, it was not possible to obtain adequate dietary records to reflect this period and we cannot objectively illustrate the increase in EA. However, the significant increase in body mass from Post XC to Pre Track (**Figure 4A**) is likely indicative of an increase in EA and a prior systematic review demonstrated female endurance athletes do not typically reduce energy intake in the non-competitive season, despite reducing total energy expenditure (23). Thus, it appears that following the cross country season, the low EA was not as severe and allowed for the increase in body mass. The increase in total body mass could also account for the increase seen in RMR, as both variables significantly increased, as did relative RMR. An increase in EA could be the reason that multiple variables improved between Post XC and Pre Track. It is interesting to note that there was a lot of agreement between the RED-S Clinical Assessment Tool and Triad Risk Classification. In all but 1 case, ‘Green’ and ‘Yellow’ in the RED-S Clinical Assessment Tool corresponded to ‘Low Risk’ and ‘Medium Risk’ respectively in the Triad Risk Classification (**Table 1**). In the 1 exception, athlete 5 was considered ‘Green’ in the RED-S Clinical Assessment Tool but ‘Moderate Risk’ in the Triad Risk Classification. Overall, both scales are extremely user friendly and the use of either scale is highly recommended in practice or in future similar research studies.

#### Limitations

While every effort was made to maintain scientific rigor, this study is not without limitations. This study used self-reported dietary intake, rather than controlling dietary intake, which could lead to measurement error. In addition, there was no second evaluation of energy availability, where dietary intake could certainly have changed over time. Due to the nature of tracking athletes in their normal environment, testing only occurred when it was convenient to incorporate within their training regimen, thus testing was not standardized to a specific menstrual phase. Finally, while the athletes were encouraged to arrive to the laboratory hydrated for body composition testing, their hydration was not confirmed by any testing.

## Conclusions

When athletes participate in competitive endurance sports, there are several factors that need to be monitored to help maintain their health and performance, particularly factors associated with RED-S. This study showed changes in body mass and body composition were not indicative of the presence of other concerns associated with RED-S, such as the observed reduced ferritin and 25(OH) vitamin D concentrations in-season, which could be impacted by micronutrient intake and thus should certainly be considered when assessing dietary habits. Therefore, there is a need for ongoing nutrition evaluation, consistent screening for ferritin throughout the competitive season, as well as a need to consider low EA as a possible cause of performance decrement if it occurs. While many studies historically have demonstrated these concerns in a controlled environment, there are few long-term prospective tracking studies in competitive athletes in this area. We propose that future research should explore these findings in larger populations of exercising women, men, and adaptive athletes to provide a new perspective on monitoring of RED-S in athletes.

## Data Availability Statement

The datasets generated for this study are available on request to the corresponding author.

## Ethics Statement

The studies involving human participants were reviewed and approved by Jacksonville University Institutional Review Board. The patients/participants provided their written informed consent to participate in this study.

## Author Contributions

All authors listed have made a substantial, direct and intellectual contribution to the work, and approved it for publication. DRH: Study design, data collection, data interpretation, manuscript preparation. JM: Study design, data collection. JTW: Study design, data interpretation, manuscript preparation. KLC: Data collection, data interpretation, manuscript preparation. GGAP: Study design data interpretation, manuscript preparation. KP: Dietary analysis, data collection. CS: Dietary analysis, data interpretation, manuscript preparation. ACH: Data interpretation, manuscript preparation. AST: Study design, data interpretation, manuscript preparation. KEA: Data interpretation, manuscript preparation.

## Conflict of Interest

The authors declare that the research was conducted in the absence of any commercial or financial relationships that could be construed as a potential conflict of interest.
